# Identifying super-feminine, super-masculine and sex-defining connections in the human braingraph

**DOI:** 10.1007/s11571-021-09687-w

**Published:** 2021-07-15

**Authors:** László Keresztes, Evelin Szögi, Bálint Varga, Vince Grolmusz

**Affiliations:** 1 PIT Bioinformatics Group, Eötvös University, H-1117 Budapest, Hungary; 2 Uratim Ltd., H-1118 Budapest, Hungary

**Keywords:** Connectome, Braingraph, SVM, Linear separation, Sex differences, Superfeminine edges, Supermasculine edges

## Abstract

For more than a decade now, we can discover and study thousands of cerebral connections with the application of diffusion magnetic resonance imaging (dMRI) techniques and the accompanying algorithmic workflow. While numerous connectomical results were published enlightening the relation between the braingraph and certain biological, medical, and psychological properties, it is still a great challenge to identify a small number of brain connections closely related to those conditions. In the present contribution, by applying the 1200 Subjects Release of the Human Connectome Project (HCP) and Support Vector Machines, we identify just 102 connections out of the total number of 1950 connections in the 83-vertex graphs of 1064 subjects, which—by a simple linear test—precisely, without any error determine the sex of the subject. Next, we re-scaled the weights of the edges—corresponding to the discovered fibers—to be between 0 and 1, and, very surprisingly, we were able to identify two graph edges out of these 102, such that, if their weights are both 1, then the connectome always belongs to a female subject, independently of the other edges. Similarly, we have identified 3 edges from these 102, whose weights, if two of them are 1 and one is 0, imply that the graph belongs to a male subject—again, independently of the other edges. We call the former 2 edges superfeminine and the first two of the 3 edges supermasculine edges of the human connectome. Even more interestingly, the edge, connecting the right Pars Triangularis and the right Superior Parietal areas, is one of the 2 superfeminine edges, and it is also the third edge, accompanying the two supermasculine connections if its weight is 0; therefore, it is also a “switching” edge. Identifying such edge-sets of distinction is the unprecedented result of this work.

**Supplementary Information:**

The online version contains supplementary material available at 10.1007/s11571-021-09687-w.

## Introduction

One of the most important challenges in brain science is establishing the cellular and anatomical causes of neurophysiological or psychological differences between human subjects. In the last decade, by the spectacular developments in magnetic resonance imaging (MRI) of the brain, together with the data-processing pipeline for the data collected, our knowledge of the cerebral connections has been increased enormously (e.g., Sporns et al. 2005; Van Essen et al. 2012; Szalkai et al. 2019a).

Diffusion MRI (dMRI) is capable of discovering the spatial anisotropy of the movement of water molecules in the brain: since in the axonal fibers of the white matter the water molecules have a diffusion movement along the axons, the axonal fibers can be tracked and traced, without any contrast material, with refined tractography algorithms (Tournier et al. 2012). With the reliable identification of the cortical- and sub-cortical gray matter areas (Fischl 2012), we can construct the connectome, or the braingraph as follows: the nodes (or vertices) of this graph are the anatomically identified gray matter areas, and two nodes are connected by an (undirected) edge if the tractography algorithm finds axonal fibers between the brain areas, corresponding to these two nodes.

Numerous results were published in the last decade, analyzing the human braingraph (Hagmann et al. 2008; Szalkai et al. 2015a; Kerepesi and Grolmusz 2017; Hagmann et al. 2012; Szalkai et al. 2019b; Craddock et al. 2013; Kerepesi et al. 2018b; Szalkai et al. 2017b; Ortiz et al. 2014). Several works describe the connections of the healthy human brain (Ball et al. 2014; Kerepesi et al. 2016; Bargmann 2012; Kerepesi et al. 2017; Batalle et al. 2013; Szalkai et al. 2017a; Kerepesi et al. 2018b; Graham 2014), while others establish relations between psychiatric diseases or conditions and the connectome (Agosta et al. 2014; Alexander-Bloch et al. 2014; Baker et al. 2014; Szalkai et al. 2019a; Besson et al. May 2014; Bonilha et al. 2014).

### Sex differences

It is known for several years that the female and the male connectomes have different properties as graphs. The work of Ingalhalikar et al. (2014) has proven—on a publicly un-available dataset—that the ratio of inter-hemispheric connections vs. the intra-hemispheric connections differs in males and females.

Our group has shown on a publicly available dataset (Kerepesi et al. 2017) that several deep graph-theoretical properties, which are usually applied in the characterization of the quality of large computer interconnection networks (Leighton 1992), are better in the braingraphs of women than in men (Szalkai et al. 2015b, 2021). We have proven that women’s braingraphs are better expanders, have greater minimal bisection width, more spanning trees, larger minimum vertex cover than that of men. In the work of Szalkai et al. (2018) we have proven that the advantage in the graph-quality parameters of women is due to the sex differences, and not to the size differences: we have compared the graphs of 36 large-brain women and 36 small-brain men, such that the brain volumes of all men were smaller than the brain volume of the smallest-brain woman in the group. We have found that men did not have better parameters than women in this test, and, additionally, many of the advantages of the women remained valid.

The adjective “better” and the noun “advantage” refer to the quality parameters of the large computer interconnection networks (Leighton 1992); their beneficial effects on the human brain functions are not proven yet (Szalkai et al. 2015b, 2021).

### Parameters, defined *a priori* versus *a posteriori*

In the studies of Ingalhalikar et al. (2014), Szalkai et al. (2015b), Szalkai et al. (2021), Szalkai et al. (2018), Fellner et al. (2020b), the authors compared parameters, which were identified *a priori*, i.e., the examination of these parameters were decided *before* the braingraphs were analyzed. In the present work, we intend to identify *a posteriori* parameters, i.e., edge-structures in the course of the analysis of the braingraphs, in which the male and female connectomes differ. Additionally, we intend to discover the smallest possible edge-sets of the braingraphs, which already determine the sex of the subject.

First, we constructed and trained a deep artificial neural network (ANN, see, e.g., Szalkai and Grolmusz 2017, 2018 for definitions and examples) for classifying the sex of the subject, using only his/her braingraph. While these efforts were moderately successful, we have found that not the deep networks, but, on the contrary, the one-level networks gave the best results for predicting the sex of the subject. In a certain sense, one-level neural networks are similar in their capabilities to linear tests or Support Vector Machines (SVMs). In the “Methods” section, we give a short introduction to SVMs.

It is important to note that we have not used artificial intelligence tools (ANNs and SVMs) for making predictors. We have used these tools for data analysis: we have found the “minimal SVM” which distinguished the sexes, then apply this SVM as a mathematical model to identify distinguished edges of the male and female connectomes.

### Few edges, which imply biological properties

It is a great challenge to identify one edge or a small set of edges in the human braingraph, which imply some important biological properties of the subject. In other words, the task is to find the most important brain connections, which relate to some biological conditions (biological property, or diseased status, or mental ability or disability). Up to now, more complex graph-theoretical properties—instead of just identifying a few graph edges—were published in this direction: for example, for the sex of the subjects, complex graph-theoretical differences were found in Szalkai et al. (2015b), Szalkai et al. (2021), Szalkai et al. (2018), Fellner et al. (2020a), Fellner et al. (2019). For intelligence-related psychological tests, some frequent neighbor sets of the hippocampus were identified, which are correlated with good and bad test results in Fellner et al. (2020b). Interrelations between graph-theoretical properties of the connectome and physiological properties were described in Szalkai et al. (2019a).

Finding one, two, or three edges whose strengths (measured in fiber numbers, cf. the “Methods” section) imply important biological properties is one of the results of the present work. These edges are the most important ones in relation to the property studied. This problem is analogous to finding the most important vertices in a graph, which was solved by Google Inc., by their famous PageRank algorithm (Page et al. 1999; Brin and Page 1998): The PageRank scoring has made the Google web search engine in front of their competitors.

Here, we identify superfeminine and supermasculine edges of the braingraph based on the largest cohort available today. These edges are described as follows.

### Few edges, which simply determine the sex of the subject

Applying Support Vector Machines and integer programming algorithms, we were able to identify a small set of connectome edges, which precisely determine the female and male brains, without any error. Additionally, and perhaps more surprisingly, we have identified 2 and 3 particular edges with the following property: if the scaled weight of both edges is 1, then the connectome belongs to a female subject. If the scaled weight of the first two of the three edges is 1, and the scaled weight of the third is 0, then the connectome belongs to a male subject. The edge weights correspond to the fiber numbers, and the scaling means that the fiber number is multiplied by an edge-specific number such that the resulting value is between 0 and 1 (the exact definition of the edge weights is given in the “Methods” section). We call these edges superfeminine and supermasculine edges, respectively.

More exactly, we are considering graphs on 83 vertices. From these 83 vertices, one can form

$$\begin{aligned} {83\atopwithdelims ()2}=3403 \end{aligned}$$

vertex-pairs, i.e., this is the maximum number of edges on 83 vertices. Note that each of the 1064 braingraphs contains exactly 83 vertices, and all of these vertices correspond to the very same 83 gray-matter areas of the brain (sometimes called ROIs, Regions of Interest).

**Fig. 1:**
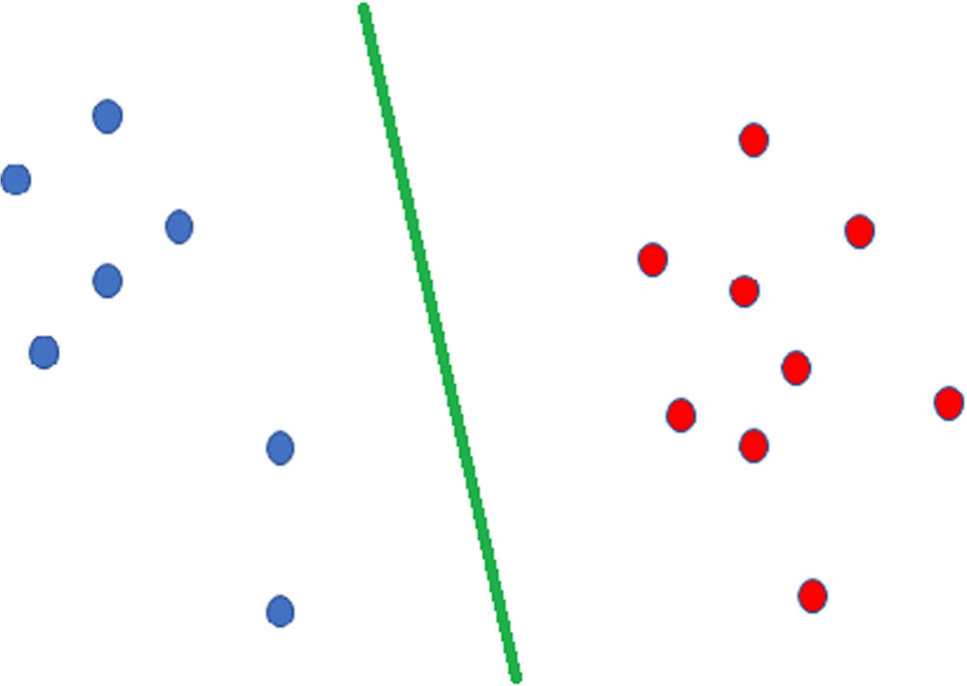
A simple example for Support Vector Machine data classification (Cortes and Vapnik 1995) on the plane. The blue and red points describe two classes of data (for example, each point corresponds to a braingraph, blue points to male, red points to female connectomes). The green line perfectly distinguishes the two classes: the blue ones are on one side, the red ones on the other side of the green line. In the 102-dimensional space (instead of the 2-dimensional space on the figure), we have succeeded in distinguishing the male and female braingraphs in a similar way: all the male graphs are on one side, all the female graphs are on the other side of our hyperplane. The coordinates of the separating hyperplane are given in the Supporting material. (Color figure online)

In our dataset of 1064 subjects, the union of all edges of the 1064 braingraphs contains 1950 edges. That means that out of the possible 3403 edges, only 1950 are present in the union of all the 1064 braingraphs. This is not a surprising observation since, for example, few areas from the left hemisphere are connected *directly* to the areas of the right hemisphere (see Supporting Fig. 1 in the on-line supporting material). As our first result, we have succeeded in finding a hyperplane in the 1950-dimensional Euclidean space, which perfectly separates the 1064 points, corresponding to the male and the female subjects (see Fig. 1). In general, it is not a great surprise: if we take an $n+1$-vertex simplex in the *n*-dimensional space, then—for any +1 and −1 labeling of those $n+1$ vertices—there exists a hyperplane which perfectly, without any error separates the −1 and the +1 labeled points as in Fig. 1. Finding a separating hyperspace in much lower dimensions is difficult and often impossible.

It is a great challenge to find the *smallest* possible set of edges, which still implies the sex of the subject. This small set of connections may carry the most important features, which differentiate the braingraphs of the sexes. If there existed a single graph edge *e* with weight *w*(*e*), such that for all braingraphs of men $w(e)>c$ and for all braingraphs for women $w(e)\le c$, then this single edge *e* would separate the sexes in a very simple way. If no such single edge exists, but there existed two edges, *e* and *f*, and three constants *a*, *b*, *c*, such that $aw(e)+bw(f)>c$ for all men and $aw(e)+bw(f)\le c$ for all women, then these two edges, *e* and *f*, would separate the sexes by a linear test. Unfortunately, no one knows one or two edges, separating the braingraphs of the sexes by simple linear tests.

We were able to identify 102 edges, which already determine the sex of the subjects (Fig. 1). Moreover, these edges determine the sex in a very simple, linear way, described below (the method of the identification of these 102 edges is detailed in the “Methods” section).

For describing this phenomenon, let us correspond each graph to a length-102 vector, with coordinates equal to the edge-weights on the chosen 102 edges. This way, we have 1064 vectors, each with 102 coordinates. In other words, we have a 102-dimensional Euclidean space with 1064 points (vectors) in it. In this space, we have determined a hyperplane, which separates the male and female graphs in the following way: all the 102-dimensional vectors made from the female graphs are on one side of the hyperplane, while all the 102-dimensional vectors, made from the male braingraphs are on the other side of the hyperplane. Consequently, (1) 102 edges out of the 1950 edges already determine the sex of the subject, and (2) in a very simple, exact, and linear way, by a separating hyperplane. Figure 1 gives a simple example for the data separation on the plane (in 2 dimensions) with a line (i.e., a line is a hyperplane on the plane).

**Fig. 2:**
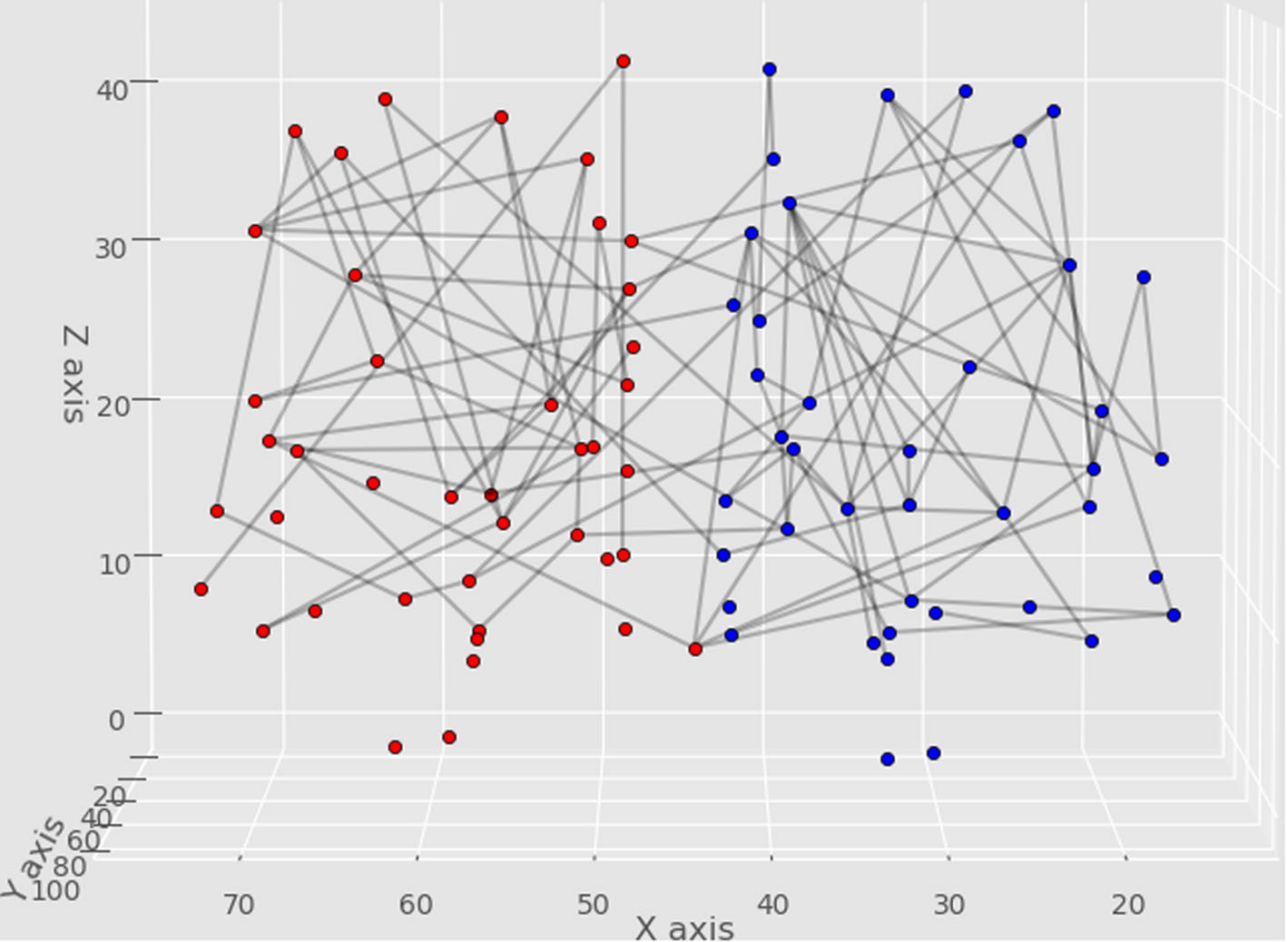
A braingraph of a subject, with 83 vertices and the 102 edges, whose weights (i.e., fiber numbers) already determine the sex of the subject. Labels on the axes are voxel coordinates in mm. In the 102-dimensional space, the male- and female braingraphs are perfectly separated by a hyperplane, similarly as the green line separates the blue and red dots in Fig. 1. The nodes from the distinct hemispheres are colored differently; the frontal lobe is on the top of the figure. The list of these 102 edges is given in Supporting Table 1 in the supporting material. An Excel file performing the actual separation-computation with all data is available at http://uratim.com/agysvm/agy-svm.zip. An interactive chart visualizing the separation can be viewed at http://pitgroup.org/static/interactive_chart/abra.html. (Color figure online)

Figure 2 depicts the 102 edges, which already determine the sex of the subject. The list of these 102 edges is given in Supporting Table 1. An Excel file performing the actual separation-computation with all data is available at http://uratim.com/agysvm/agy-svm.zip. An interactive chart visualizing the separation can be viewed at http://pitgroup.org/static/interactive_chart/abra.html

### Superfeminine and supermasculine edges

Our second main result is the identification of very few connections, out of the 102 edges, in a way that if each of these edges has specific (either high or low) weights, then the sex of the subject is uniquely determined.

Let us recall that the weight of an edge is the number of the axonal fibers found running between its two endpoints in the tractography algorithm, scaled for individual edges to be between 0 and 1 (the details are given in the “Methods” section).

We have found that if the weights of both edges below are 1, then, independently from the weights of the remaining 100 edges out of the 102 sex-determining connections, the sex of the subject is female:

F1: (rh.superiorfrontal, Left-Putamen)

F2: (rh.parstriangularis, rh.superiorparietal)

We call the set of edges F1. F2 “superfeminine” edges.

Similarly, we have found three edges, such that, if the weights of the first two are high and the weight of the third one is low, then, independently of the other edge-weights of the remaining 99 edges out of the 102 connections, the sex of the subject is male:

M1: (1h.rostralmiddlefrontal, Left-Thalamus-Proper)

M2: (Right-Hippocampus, lh.supramarginal)

F2: (rh.parstriangularis, rh.superiorparietal)

**Fig. 3:**
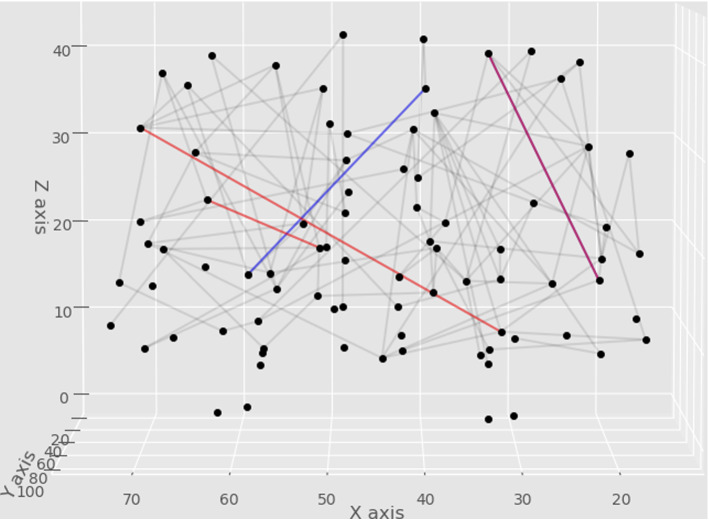
The superfeminine (blue) and supermasculine (red) edges, and the switching edge (purple). If the fiber numbers of both the blue edge and the purple edge are high, then the subject is female. If the fiber number of both the red edges are high and the purple edge is low, then the subject is male, independently of the fiber numbers of the other edges. (Color figure online)

The superfeminine and supermasculine edges are depicted in Fig. 3.

We call edges M1 and M2 “supermasculine” edges. Note that edge F2 is present in both sets: if the weight of F1 and F2 are high, then it implies that the graph belongs to a female subject, and if the weight of F2 is low, and the weights of M1 and M2 are high, then the graph belongs to a male subject. We call the edge F2 a “switching” edge. We refer to the exact definition of the “switching” edge in the “Methods” section.

## Methods

### Graph construction

Our data source is the 1200 Subjects Release of the Human Connectome Project (HCP) (McNab et al. 2013), available at the https://www.humanconnectome.org site. The subjects were healthy adults between 22 and 35 years of age. The data acquisition methodology of the Human Connectome Project is detailed in the “WU-Minn HCP 1200 Subjects Data Release: Reference Manual” at the site https://www.humanconnectome.org/storage/app/media/documentation/s1200/HCP_S1200_Release_Reference_Manual.pdf.

We have applied the 3T MR diffusion imaging data and processed it with the Connectome Mapper Tool Kit (CMTK) (Daducci et al. 2012).

Our goal was the construction of graphs, or connectomes, which describe the connections between the distinct, anatomically identified cortical and sub-cortical, gray-matter areas of the brain of the subjects. The nodes (or vertices) of our graphs corresponded to the anatomically identified gray matter areas, and we connected two nodes by an edge if the workflow described below found axonal fibers running between the areas that corresponded to the nodes. We emphasize that the study of the connectome instead of the whole MR image deals with *exclusively* the connections between the gray matter areas and does not take into account the exact orbit of the axonal fibers running in the white matter of the brain. This way, we can work with graphs instead of very redundant spatial imagery gained from the processing of the diffusion MR images. We note that the (mathematical) graph theory, which was established in 1741 by a work of Euler (1741), has very rich structures and several of the most complex and deepest proofs and tools in mathematics (e.g., Szemeredi 1975; Chudnovsky et al. 2006; Erdos et al. 1946). Therefore, the transition from images to graphs facilitates the application of the well-developed techniques of the (mathematical) graph theory to one of the most complex organs on Earth, the human brain.

The axonal fibers are discovered from the diffusion MR images by tractography algorithms. Probabilistic tractography was applied, with 1 million streamlines, by using MRtrix 0.2 tractography software. For each subject, the tractography program was run 10 times. In each run, the number of fibers was determined for each edge. If in any of the ten runs an edge was non-existent, that is, it was not defined by any fiber in the tractography, then that edge was discarded. Next, from these 10 runs, for each edge, the maximum and minimum numbers of fibers were deleted, and the average of the remaining 8 fiber numbers was assigned to the edge; this number is used as the weight of the edge. This way, the false positive and false negative edges were dealt with, and large errors, leading to the maximum or minimum fiber numbers of an edge, were discarded: they did not influence the average value (Varga and Grolmusz 2021).

For each subject, 5 graphs, each with resolutions of 83, 129, 234, 463, and 1015 nodes were computed, by applying the CMTK’s implementation of the FreeSurfer suite of programs for parcellation (Fischl 2012; Desikan et al. 2006; Tournier et al. 2012).

The HCP public release contains the data of 1206 subjects. From these, 1113 contained structural scans. Our workflow (Varga and Grolmusz 2021) was successfully completed for the data of 1064 subjects. From the subjects, there were 575 females and 489 males. The resulting graphs, with 5 resolutions for each subject, can be downloaded from the site http://braingraph.org/download-pit-group-connectomes/. For the detailed description of the graph-constructing workflow and the resulting graph dataset, we refer to the publication (Varga and Grolmusz 2021).

In the present work, we apply only the coarsest 83-node resolution, i.e., we consider 1064 graphs of 1064 subjects, each on 83 vertices. We have found 1950 edges by taking the union of the edges of the 1064 braingraphs on 83 vertices.

In braingraph *u* the edge *v* is denoted by $e^u_v$, for $u=1,2,\ldots ,1064$, $v=1,2,\ldots ,1950$. The weight of the edge $e^u_v$, denoted by $w(e^u_v)$, is the average number of axonal fibers found running between its endpoints in the 8 tractography computations.

### An edge-specific weight-scaling method

We would like to scale individually the weights of the edges such that all the resulting edge-weights are between 0 and 1, as follows:

1 $$\begin{aligned} y_{i}^\ell :=\frac{w(e_{i}^\ell ) - \min \limits _{u=1}^k w(e_{i}^u) }{\max \limits _{u=1}^k w(e_{i}^u) - \min \limits _{u=1}^k w(e_{i}^u)} \end{aligned}$$

if the denominator is not zero; otherwise, let $y_{i}^\ell$ be zero; $k=1064$. This way, for each braingraph, and for each edge, the smallest weight is transformed to 0, and the largest (if differs from the smallest) to 1. From now on, we use this scaled weights $y_i^\ell$, instead of the original ones. Let $y^\ell =(y_{1}^\ell ,y_{2}^\ell ,\ldots ,y_{s}^\ell ), s=1950$.

In other words, for any $\ell$, $y^\ell$ describes a braingraph, with the new, scaled edges as its coordinates.

In what follows, we do not use the superscript $\ell$ if the meaning of *x* is clear from the context.

### An SVM-based technique with heuristic improvements

The support vector machines (SVMs) are frequently used tools in artificial intelligence to classify the elements of large data sets (Cortes and Vapnik 1995).

Suppose that we have *k* data points $x^1,x^2,\ldots , x^k$ in the *n*-dimensional Euclidean space $\mathbb {R}^n$, and a function $f:\mathbb {R}^n\rightarrow \{0,1\}$. We intend to find an *n*-dimensional hyperplane, such that

1. one side of the hyperplane contains all $x^i$’s with $f(x^i)=1$, and the other side of the hyperplane contains all $x^j$’s with $f(x^j)=0$, and
2. the hyperplane separates the data points with the largest margin, that is, the distance of the closest data point to the hyperplane is maximized.

If $n\ge k$ then the requirement (1) can always be met if the $x^1,x^2,\ldots , x^k$ points are in a general position in the *n*-dimensional Euclidean space $\mathbb {R}^n$ (one can see this simply by solving a linear system of equations with a non-zero determinant for finding the normal vector of the hyperplane). If $n<k$, then (1) (i.e., the perfect separation with a hyperspace) is not always satisfiable. We refer to Cover’s theorem for probability estimations for the satisfiability of (1) when $n<k$ (Cover 1965).

In the present work, first, we solved (1) and (2) for the $n=1950$ dimensional space, with $k=1064$, by using the Python Scikit-Learn suite (Hao and Ho 2019). Next, we intend to reduce the coordinates (i.e., the number of edges), which are present in the separation. In other words, we needed to find as few coordinates as possible, such that the male and female connectomes can be separated by a hyperspace, using only the chosen coordinates.

This goal can be formalized as follows:

Let $\Vert w\Vert _0$ denote the number of the non-zero coordinates of vector *w*. Then we need to find

2 $$\begin{aligned} \min \Vert w\Vert _{0}, \end{aligned}$$

satisfying

3 $$\begin{aligned} w\cdot x + b \ge 0\quad \hbox {for all } x, \end{aligned}$$

corresponding to a female braingraph, and

4 $$\begin{aligned} w\cdot x + b < 0\quad \hbox {for all }x, \end{aligned}$$

corresponding to a male braingraph.

By the best of our knowledge, no optimization method is known for solving this problem exactly in polynomial time. Here we have applied the combination of two simple heuristic solution methods, by which we were able to reduce $\Vert w\Vert _{0}$ from 1950 to 102. In other words, we can identify 102 coordinates of $x=y$ or, equivalently, 102 edges of the graph, such that the sex of the corresponding subject can be expressed by the sign of the linear expression $w\cdot x + b$ . The value of *b* and the 102 non-zero coordinates of *w* are given in the Supporting material, in Supporting Table 2.

The first heuristic algorithm is a Weight-Based Dimension-Reduction Algorithm (WBDRA): Here, we start with a *w*, which separates linearly, and next delete the smallest weight coordinates of *w*. A rate parameter *r* defines that the *r* fraction of the smallest coordinates needs to be deleted. If the new *w* does not separate, then we backtrack and decrease *r*. The code of the algorithm is given in the Supporting Material, as Program Code 1.

The second procedure is a Single Dimension Deleting Algorithm (SDDA): Here, we start with a separating *w*, and take a random order of the non-zero coordinates of *w*, and attempt to delete one dimension if the separation property remains valid. If not, then we try to delete the next dimension. The code of SDDA is given as Program Code 2 in the Supporting Material.

With the application of the two heuristic algorithms (WBDRA, SDDA), we have succeeded in reducing the $\Vert w\Vert _{0}$ to 102.

We need to add that we cannot prove the optimality of the 102-dimensional solution: we think that even better results can be reached. However, by using Cover’s theorem (Cover 1965), the probability that randomly 0–1 labeled, randomly chosen $k=1064$ points are separable by a hyperplane in 102 dimensions is much less than $2^{-100}$.

Since our data points are not randomly distributed, we intended to investigate the specialty of the existence of the 102-dimensional SVM for our 1064 data points.

We have focused on our main tool, the WBDRA algorithm: using only this procedure, we were able to identify a 115-dimensional sex-separating weight vector—by using SSDA—this dimension was reduced to 102. Since the WBRDA algorithm is much faster than the SSDA, we used WBRDA in the tests below.

We have performed the following tests 50 times for our specific data points:

- We assigned randomly 575 1-labels, and 489 0-labels to the 1064 data points *y*, corresponding to weighted-edge brain graphs;
- next, we have applied the WBRDA algorithm.

The smallest dimension we find was 223, the largest 293, the average 256.6. Therefore, the 115-dimensional separation of the sexes, found by using the WBRDA algorithm exclusively is a surprising result, even for the specific *y* points, representing our 1064 braingraphs.

### Finding superfeminine and supermasculine edges

Our goal is to identify edges, which have the greatest impact on decisions (3) and (4). These edges may have very important roles in the sex-specific development and functioning of the human brain. Simply stated, the most important edges would have the coordinates with the largest absolute values in vector *w* in (3) and (4). In what follows, we formally define 0-generator and 1-generator coordinates for a given function $f:[0,1]^N\rightarrow \{0,1\}$; in our application *f* maps weighted edge-sequences to the sex of the subject.

Let [*N*] denote the set $\{1, 2\dots N\}$.

For $y \in [0, 1]^{N}$ and $I \subset [N]$ let $y|_{I}\in [0, 1]^{N}$ denote:

$$\begin{aligned} y|_{I}(j)= {\left\{ \begin{array}{ll} y_{j} & \text { if }j \in I \\ 0 & \text { otherwise. } \end{array}\right. } \end{aligned}$$

Let $\mathcal {G}$ denote the set of our 1064 braingraphs, each represented by an $x\in [0,1]^N$; originally, $N=1950$, i.e., each braingraph was represented by a 1950 weighted edges. In the previous section, we have seen that we can reduce $N=102$.

For an $I \subset [N]$ let $\mathcal {G}|_{I}:=\{x|_{I}: x \in \mathcal {G}\}$.

#### **Definition 1**

We say that $I \subset [N]$ is a 1-generator for *f* with a seed $x\in [0,1]^{N}|_{I}$, if $\forall y \in \mathcal {G}|_{[N]-I}$ $f(x+y) = 1$. Similarly, we say that $I \subset [N]$ is a 0-generator for *f* with a seed $x\in [0,1]^{N}|_{I}$ if $\forall y \in \mathcal {G}|_{[N]-I}$ $f(x+y) = 0$ is satisfied.

In other words, the seed values in the coordinates in the 0-generator or 1-generator *I* already determine the value of our *f*.

Our goal is finding the smallest 0- and 1-generators for *f*, where *f* gives the sex of the subject: $f(x)=0$ for males, and $f(x)=1$ for females:

$$\begin{aligned} f(x)= {\left\{ \begin{array}{ll} 1 & \text { if }w\cdot x + b \ge 0 \\ 0 & \text { if }w\cdot x + b < 0 \end{array}\right. } \end{aligned}$$

For this *f*, finding the minimal 0- and 1-generators is essentially a version of a knapsack problem, solvable by integer programming methods. For the reduction, we need some definitions and simple statements:

#### **Definition 2**

For any fixed $w\in [0,1]^N$, let $z_{F} \in [0, 1]^{N}$ be defined

$$\begin{aligned} z_{F}(i)= {\left\{ \begin{array}{ll} 1 & \text { if }w_{i} \ge 0 \\ 0 & \text { if }w_{i} < 0 \end{array}\right. } \end{aligned}$$

Let $z_{M} \in [0, 1]^{N}$ be defined

$$\begin{aligned} z_{M}(i)= {\left\{ \begin{array}{ll} 1 & \text { ha }w_{i} \le 0 \\ 0 & \text { ha }w_{i} > 0 \end{array}\right. } \end{aligned}$$

It is easy to see that $x=z_F$ maximizes and $x=z_M$ minimizes $w\cdot x + b$.

We show the reduction for 1-generators; for 0-generators a similar reduction works.

#### **Lemma 1**

*If* $I \subset [N]$ *is a* 1-*generator for* *f* *with seed* $x\in [0,1]^{N}|_{I}$ *then it is also a* 1-*generator with seed* $z_{F}|_{I}$

#### *Proof*

Let $$y \in \mathcal {G}|_{[N]-I}$$, then $$w\cdot (z_{F}|_{I}+y) + b \ge w\cdot (x+y) > 0$$. $$\square$$

The next Corollary is obvious:

#### **Corollary 2**

*If* *I* *the smallest* 1-*generator with any seed, then it is also the smallest* 1-*generator with seed* $z_{F}|_{I}$. $\square$

#### **Lemma 3**

*Let* $\xi _{i}$ *denote the coordinates of the* 0–1*characteristic vector of set* *I*: $\xi _{i}=1$ *if and only if* $i\in I$. *Then* *I* *is a* 1-*generator for* *f* *with a seed* $z_{F}|_{I}$ *if and only if* $\forall x \in \mathcal {G}$, $f(x)=0$ *implies* $\sum _{i=1}^{N}\xi _{i}\cdot w_{i}(z_{F}(i)-x_{i}) > -w\cdot x - b$.

#### *Proof*

5 $$\begin{aligned} \sum _{i=1}^{N}\xi _{i}\cdot w_{i}(z_{F}(i)-x_{i}) + w\cdot x + b = w\cdot (z_{F}|_{I}+x|_{[N]-I}) + b \end{aligned}$$

is non-negative. $\square$

Note that for any $x: f(x)=1$, the (5) is also non-negative.

From Lemma 3, the optimization problem, which gives the minimum 1-generator, can be written: Minimize $\sum _{i=1}^{N}\xi _{i}$, with the condition that for all $x\in \mathcal {G}:$

$$\begin{aligned} \sum _{i=1}^{N}\xi _{i}\cdot w_{i}(z_{F}(i)-x_{i}) \ge -w\cdot x - b. \end{aligned}$$

#### **Definition 3**

The edges in 1-generators, where the corresponding $z_F$ seed coordinates are ones, are called superfeminine edges. The edges in 0-generators, where the corresponding $z_M$ seed coordinates are ones, are called supermasculine edges. An edge *e* is called a switching edge, if any of the following two properties holds for it:

1. *e* is a superfeminine edge and it is also in a 0-generator with the corresponding $z_M$ seed coordinate 0; or
2. *e* is a supermasculine edge, which is also in a 1-generator with the corresponding $z_F$ seed value 0.

The distinction of by the 1 seed-coordinates are made since the weights correspond to fiber numbers, and the “strong” graph edges, defined by many fibers, are called superfeminine or supermasculine edges; we do not intend to call “weak” edges, i.e., edges with the fewest fiber tracts superfeminine or supermasculine, even if they are the part of a 0- or 1-generator (e.g., we do not call F2 a supermasculine edge). The superfeminine and supermasculine edges we found are depicted on Fig. 3.

### Software used

The braingraphs were computed by using the CMTK suite (Daducci et al. 2012), with the details given in the beginning of the section. The figures were created by using Python Matplotlib mplot3D and Networkx packages. The 1950-dimensional SVM was computed using the Python Scikit-Learn suite of programs (Hao and Ho 2019). The heuristic improvements, resulting in the 102-dimensional separation, were found by the programs given in the Supporting Material in the Program codes section. For IP optimization, we used the Python Pulp package.

## Discussion and results

Most cerebral sex dimorphism studies to date were done on very small (up to 40–80 subjects) cohorts and applied mostly volumetric investigations (Frederikse et al. 1999; Koscik et al. 2009; Maleki et al. 2012; Butler et al. 2006). Our previous works (Szalkai et al. 2015b, 2021, 2018; Fellner et al. 2019, 2020a, 2020c) first demonstrated sex dimorphisms in *a priori* defined graph parameters; in most cases the better connectivity-related parameters were found in the female connectomes.

Here we first demonstrate relatively small edge-sets, which determine the sex of the subjects on a very large, 1064-member cohort.

The 102 edges, which already define the sex of the subjects – without any error—are listed in the Supporting material as Supporting Table 1. Obviously, numerous edges connect subcortical nuclei with other parts of the brain. 13 of these 102 edges are inter-hemispheric.

The most frequently appearing nodes in these 102 edges, without considering lateralization, are the inferiorparietal (10 times), posteriorcingulate (9 times), precuneus (9 times), superiorparietal (8 times).

It is known that the inferior parietal lobule, which is a part of the heteromodal association cortex (HASC), shows sexual volumetric dimorphisms (Frederikse et al. 1999; Koscik et al. 2009).

The sex differences in the development of migraine and the role of precuneus were reported in Maleki et al. (2012) and in mental rotation (Butler et al. 2006).

Counting with lateralization, the most frequent nodes are the rh.precuneus (7 times), rh.inferiorparietal (6 times), rh.posteriorcingulate (6 times) and the right-pallidum (6 times), all in the right hemisphere.

To the best of our knowledge, we are the first showing that not only these nodes of the braingraph, but rather their important connections, listed in Supporting Table 1, carry substantial sex dimorphisms.

Additionally, we are the first to show the existence of superfeminine and supermasculine edges.

The superfeminine edges we have found are

F1: (rh.superiorfrontal, Left-Putamen)

F2: (rh.parstriangularis, rh.superiorparietal).

The two supermasculine edges with the F2 “switching” edge are:

M1: (1h.rostralmiddlefrontal, Left-Thalamus-Proper)

M2: (Right-Hippocampus, lh.supramarginal)

F2: (rh.parstriangularis, rh.superiorparietal)

The weights in fiber numbers of these edges are between 0 and 13.5 for F1; 0 and 385.375 for F2; 0 and 2010.5 for M1 and 0 and 27 for M2. Note that the weights are computed for each edge as the average of 8 tractography runs; therefore, they are not always integers.

The most interesting edge is F2, which, with high weight, is a superfeminine edge, and with low weight, and with M1 and M2 with high weights, it implies the male sex of the subject. We note that we use the terms “high” and “low” here, instead of 1 and 0 here. This is because if we set the weight of F1 and F2 both to 1, then the test will decide that the subject is a female (see Corollary 2); but it may happen that no actual female braingraph has the weight of F1 and F2 equal to exactly 1.

The area of Pars Triangularis was related to hormonal (oxytocin and arginine vasopressin) effects in men, and the same hormones to the parietal cortex—instead of Pars Triangularis—in women (Rubin et al. 2017). It is striking that just this edge, connecting the Pars Triangularis and the Superior Parietal area in the right hemisphere, has this distinguished “switching” property. Other publications also report sex differences in Pars Triangularis and the parietal cortex in context with hormonal regulation (Striepens et al. 2014; Hecht et al. 2017; Skvortsova et al. 2020), speech-language production (Foundas et al. 1998; Frederikse et al. 1999; Yao et al. 2020), in mental rotation performance (Koscik et al. 2009).

There exist numerous other sets of edges with the superfeminine and supermasculine property; we demonstrated these since they were the smallest set we have found. We note that knowing *only* the weights of F1, F2 or M1, M2 and F1 will not imply the sex in general; except when their weights are extremal.

## Conclusions

Instead of “a priori” hypotheses, we have followed an “a posteriori” way of search for edges in the human connectome, which determine the sex of the subjects. We have identified 102 edges that determine the sex in a very simple, linear way in a 1064-member cohort. Instead of considering all the possible 1950 edges, only these 102 edges imply the sex of the subject without any error.

First in the literature, we have found two and three edges, out of the 102 ones, whose weights being properly set, imply the sex of the subject, independently of the other edges in the graph. The right Pars Triangularis area is present as an endpoint in these edges. This area is related to hormonal (oxytocin and arginine vasopressin) effects in men and the same hormones to the parietal cortex – instead of Pars Triangularis—in women (Rubin et al. 2017). The parietal cortex is also present as an endpoint in these edges.

The novel edge-specific scaling of the weights of the edges, given by the formula (1), contributed to the definition of the superfeminine and the supermasculine edges.

## Supplementary Information

Below is the link to the electronic supplementary material.

Supplementary file1 (PDF 358 kb)

## Data Availability

The data source of this work was published at the Human Connectome Project’s website at http://www.humanconnectome.org (McNab et al. 2013) as the 1200-subjects public release. The parcellation data, containing the anatomically labeled ROIs, is listed in the CMTK nypipe GitHub repository https://github.com/LTS5/cmp_nipype/blob/master/cmtklib/data/parcellation/lausanne2008/ParcellationLausanne2008.xls. The large Excel table, which computes the linear separation of the 1064 male and female braingraphs, using only 102 edges, can be accessed at http://uratim.com/agysvm/agy-svm.zip; note that in the Excel file the non-scaled weights are present, to facilitate easy verification. The interactive chart showing the linear separation between the 1064 braingraphs of the sexes is available at http://pitgroup.org/static/interactive_chart/abra.html.
