# Supplementary material for: Identifying super-feminine, super-masculine and sex-defining connections in the human braingraph
Source: Cogn Neurodyn. 2021 Jul 15;15(6):949–59. doi: 10.1007/s11571-021-09687-w (PMC8572280; doi:10.1007/s11571-021-09687-w)
Supplement: Supplementary file 1 — Supplementary file1 (PDF 358 kb) [file 11571_2021_9687_MOESM1_ESM.pdf]

Supporting Material for the Work: Identifying Super-Feminine, Super-Masculine and Sex-Defining Connections in the Human Braingraph

László Keresztes[a,**], Evelin Szögi[a,**], Bálint Varga[a], Vince Grolmusz[a,b,*]

[a] *PIT Bioinformatics Group, Eötvös University, H-1117 Budapest, Hungary*
[b] *Uratim Ltd., H-1118 Budapest, Hungary*

---

[*] Corresponding author
[**] Joint first authors
Email addresses: keresztes@pitgroup.org (László Keresztes), szogi@pitgroup.org (Evelin Szögi), balorkany@pitgroup.org (Bálint Varga), grolmusz@pitgroup.org (Vince Grolmusz)

Supporting Figure

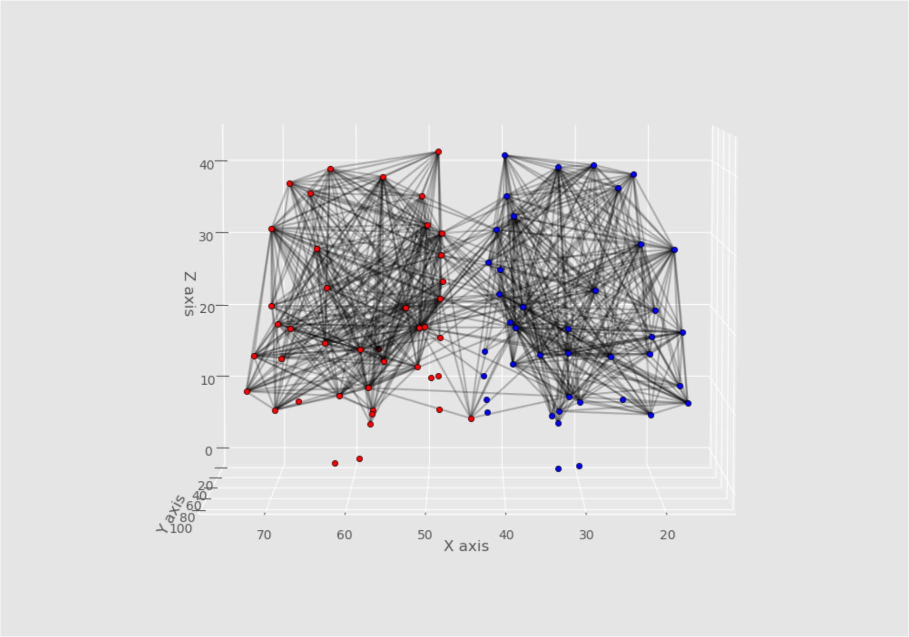

Supporting Figure 1: The braingraph of the subject of ID number 100206, with 83 vertices. The nodes from the left hemisphere are colored red, the ones from the right hemisphere are colored blue.

Supporting Tables

Supporting Table 1

Here we list the 102 edges we have identified characterizing the sex of the subjects. The ROIs are named according to the table given in `https://github.com/LTS5/cmp_nipype/blob/master/cmtklib/data/parcellation/lausanne2008/ParcellationLausanne2008.xls`.

1 (rh.precuneus, Right-Hippocampus)
2 (rh.caudalmiddlefrontal, Left-Hippocampus)
3 (rh.parahippocampal, Right-Thalamus-Proper)
4 (rh.parsopercularis, rh.rostralmiddlefrontal)
5 (rh.posteriorcingulate, rh.insula)
6 (rh.posteriorcingulate, rh.bankssts)
7 (lh.paracentral, Left-Accumbens-area)
8 (rh.precuneus, rh.pericalcarine)
9 (lh.rostralmiddlefrontal, Left-Thalamus-Proper)
10 (rh.insula, Right-Pallidum)

```
(rh.posteriorcingulate, Brain-Stem)
(rh.inferiorparietal, rh.transversetemporal)
(rh.lingual, lh.lingual)
(lh.superiorparietal, Left-Caudate)
(rh.precentral, lh.posteriorcingulate)
(rh.parahippocampal, rh.middletemporal)
(lh.rostralmiddlefrontal, lh.postcentral)
(lh.precuneus, lh.pericalcarine)
(rh.caudalanteriorcingulate, rh.posteriorcingulate)
(lh.parsopercularis, lh.rostralmiddlefrontal)
(lh.fusiform, lh.superiortemporal)
(rh.inferiorparietal, rh.precuneus)
(rh.superiorfrontal, Left-Putamen)
(lh.caudalmiddlefrontal, Left-Pallidum)
(lh.bankssts, Left-Caudate)
(rh.paracentral, rh.cuneus)
(rh.supramarginal, rh.bankssts)
(rh.inferiorparietal, rh.insula)
(rh.precentral, Brain-Stem)
(Right-Hippocampus, Brain-Stem)
(rh.lingual, Right-Thalamus-Proper)
(lh.isthmuscingulate, lh.inferiorparietal)
(rh.lateraloccipital, Right-Putamen)
(lh.isthmuscingulate, lh.precuneus)
(rh.pericalcarine, rh.lingual)
(Right-Hippocampus, lh.supramarginal)
(lh.postcentral, lh.superiortemporal)
(rh.superiorparietal, rh.inferiorparietal)
(lh.superiorparietal, Left-Thalamus-Proper)
(lh.supramarginal, lh.superiorparietal)
(rh.superiorparietal, Right-Caudate)
(rh.middletemporal, Right-Hippocampus)
(lh.pericalcarine, lh.inferiortemporal)
(lh.posteriorcingulate, lh.supramarginal)
(lh.transversetemporal, Left-Thalamus-Proper)
(rh.precentral, rh.middletemporal)
(rh.precentral, rh.postcentral)
(rh.rostralmiddlefrontal, lh.posteriorcingulate)
(rh.isthmuscingulate, rh.pericalcarine)
(lh.superiorparietal, lh.inferiorparietal)
(rh.caudalanteriorcingulate, lh.parsopercularis)
(lh.inferiorparietal, lh.bankssts)
(rh.parstriangularis, rh.parsopercularis)
(rh.insula, Brain-Stem)
(rh.precuneus, Right-Putamen)
(lh.paracentral, lh.middletemporal)
```

57 (rh.parstriangularis, rh.superiorparietal)
58 (lh.inferiortemporal, Left-Pallidum)
59 (rh.postcentral, rh.transversetemporal)
60 (lh.caudalanteriorcingulate, Left-Pallidum)
61 (rh.isthmuscingulate, Right-Caudate)
62 (lh.fusiform, Left-Hippocampus)
63 (Left-Caudate, Left-Putamen)
64 (rh.lateralorbitofrontal, Right-Pallidum)
65 (rh.superiorparietal, rh.bankssts)
66 (lh.precentral, Left-Putamen)
67 (lh.bankssts, Brain-Stem)
68 (rh.precuneus, rh.lateraloccipital)
69 (lh.caudalanteriorcingulate, lh.inferiorparietal)
70 (Right-Putamen, Right-Accumbens-area)
71 (lh.lingual, lh.parahippocampal)
72 (Right-Pallidum, lh.caudalmiddlefrontal)
73 (Right-Thalamus-Proper, Right-Pallidum)
74 (rh.superiorfrontal, rh.paracentral)
75 (rh.rostralanteriorcingulate, Right-Thalamus-Proper)
76 (lh.lateraloccipital, lh.bankssts)
77 (lh.caudalanteriorcingulate, Left-Caudate)
78 (rh.supramarginal, rh.transversetemporal)
79 (lh.superiorfrontal, lh.supramarginal)
80 (lh.cuneus, Left-Pallidum)
81 (rh.fusiform, rh.inferiortemporal)
82 (rh.inferiorparietal, Right-Pallidum)
83 (rh.rostralmiddlefrontal, Right-Putamen)
84 (lh.superiorfrontal, lh.lateraloccipital)
85 (rh.medialorbitofrontal, rh.parstriangularis)
86 (lh.precentral, lh.supramarginal)
87 (rh.transversetemporal, Right-Hippocampus)
88 (Right-Thalamus-Proper, lh.lateraloccipital)
89 (rh.posteriorcingulate, lh.caudalanteriorcingulate)
90 (rh.inferiorparietal, Left-Hippocampus)
91 (Right-Accumbens-area, lh.precentral)
92 (rh.pericalcarine, rh.transversetemporal)
93 (lh.parahippocampal, lh.transversetemporal)
94 (rh.posteriorcingulate, rh.isthmuscingulate)
95 (rh.rostralanteriorcingulate, Right-Caudate)
96 (lh.lingual, Left-Thalamus-Proper)
97 (rh.postcentral, rh.cuneus)
98 (rh.caudalmiddlefrontal, Right-Pallidum)
99 (lh.postcentral, Left-Pallidum)
100 (lh.superiorfrontal, Left-Caudate)
101 (rh.precuneus, Right-Amygdala)
102 (rh.precuneus, rh.inferiortemporal)

Supporting Table 2

Here we list the numerical values of the coefficients of the linear expression $w \cdot x + b$, which satisfies

$$w \cdot x + b > 0$$

for all x, corresponding to a female braingraph, and

$$w \cdot x + b < 0$$

for all x, corresponding to a male braingraph.

The number $b = -6.038549870659588237e+01$. The coordinates of the 102-dimensional vector w, in the same order as the edges are listed in Supporting Table 1:

```
1  3.140827577736224896e+01
2  -4.224516926777828019e+01
3  -5.534731949947487095e+01
4  -5.021383798706737167e+01
5  3.338868535424175121e+01
6  -6.441754383644450854e+01
7  -9.137204014740284208e+00
8  -1.623199041116811969e+01
9  -1.404986301933692516e+02
10 -4.006330872545306221e+01
11 -4.252783335733649039e+01
12 4.178817508618046617e+01
13 4.372649839601213984e+01
14 4.490601147649471869e+01
15 4.707127337369107067e+01
16 -3.636412443899504154e+01
17 7.015011711412499551e+01
18 4.336665095022959093e+01
19 -7.814457117294969635e+01
20 -4.214089662663334934e+01
21 2.128444054185646195e+01
22 3.312373784233776774e+01
23 1.639952878714990732e+02
24 3.026734348412119502e+01
25 6.268856306230090070e+01
26 -4.715689733082451340e+01
27 2.432515011860931509e+01
28 -3.448165961059984852e+01
29 3.340173045666283969e+01
30 4.042781804276076230e+01
31 2.761020298703500586e+01
32 2.402973906860708198e+01
```

```
5.799632593673756986e+01
2.811207336490517150e+01
-3.530994163115634166e+01
-1.569561312762774605e+02
7.724860503993652117e+01
-2.206056231378179433e+01
2.931487757932982774e+01
-2.102330615674154757e+01
2.327980794756512850e+01
7.887033703905483151e+01
-1.652915811375330790e+01
2.233205680678592842e+01
-3.287713377723386543e+01
3.098515708552118397e+01
4.994991466114837664e+01
-1.208699629202809689e+01
-3.915248510214356514e+01
-3.627962519972498256e+01
-2.723952954348446909e+01
-4.192812642391789524e+01
-2.172699471903166213e+01
-5.976128513966504840e+01
6.259311751749017816e+01
3.402550885586439477e+01
2.210190354984765690e+02
-3.676412998508114072e+01
-8.721544084472516545e+01
3.913733184781763441e+01
3.834602562855815222e+01
3.638168347536320368e+01
4.718280018698974487e+01
3.251867640180642383e+01
4.918992981007945531e+01
-2.066292878945655787e+01
-4.066693930915500488e+01
-2.157875932254564333e+01
7.650679496763183352e+01
4.825588522618672727e+01
4.970415751004980365e+01
7.295806396686961648e+01
-2.882074964324494104e+01
3.025214670489702584e+01
7.392751412897777641e+01
3.849139711312415812e+01
-2.672208728717341941e+01
-7.607620421231430896e+01
```

```
-6.393366605226245980e+01
2.138202760440539052e+01
-3.614660559000751761e+01
2.635867128635015533e+01
7.575343108027709604e+01
4.491884096447378738e+01
9.919165525049973553e+01
2.689606565844382047e+01
-4.580873022162855079e+01
3.428996344958203935e+01
2.134704175565022766e+01
-1.729583819036137982e+01
5.079962213472451538e+01
5.934224772754128452e+01
5.915239459823803259e+01
1.812222940561209938e+01
5.407256405097979979e+01
4.579401150729591308e+01
7.263014051692987039e+01
2.971896563339183928e+01
6.289375521391245627e+01
-4.140460314388891305e+01
-4.235611871509448179e+01
4.713752575052093619e+01
```

Program Codes

Program Code 1

```
def drop_by_weight(features, rate=0.2, rate_divisor=1.2):
    tmp_features = features
    while True:
        x_ = x[:, tmp_features]
        clf = LinearSVC(random_state=0, tol=1e-5)
        clf.fit(x_, y)
        y_clf = clf.predict(x_)
        if np.array_equal(y, y_clf):
            features = tmp_features
            w_abs = np.sort(np.abs(clf.coef_[0]))
            threshold = w_abs[int(rate * len(w_abs))]
            tmp_features = [f for f in features if
                  np.abs(clf.coef_[0, features.index(f)]) > threshold]
        else:
            rate_threshold = float(1)/len(features)
            if rate > rate_threshold:
                tmp_features = features
```

```
            rate = max(float(rate)/rate_divisor, rate_threshold)
        else:
            break

    return features
```

Program Code 2

```
def drop_one(features):
    while True:
        fl = len(features)
        random.shuffle(features)
        for feature in features:
            clf = LinearSVC(random_state=0, tol=1e-5)
            tmp_features = list(features)
            tmp_features.remove(feature)
            clf.fit(x[:, tmp_features], y)
            y_clf = clf.predict(x[:, tmp_features])
            if np.array_equal(y, y_clf):
                features.remove(feature)
                break
        if len(features) == fl:
            break
    return features
```
